# The role of CD28 in the prognosis of young lung adenocarcinoma patients

**DOI:** 10.1186/s12885-020-07412-0

**Published:** 2020-09-23

**Authors:** Dantong Sun, Lu Tian, Tiantian Bian, Han Zhao, Junyan Tao, Lizong Feng, Qiaoling Liu, Helei Hou

**Affiliations:** 1 Precision Medicine Center of Oncology, the Affiliated Hospital of Qingdao University, No. 59 Haier Road, Qingdao, 266000 Shandong China; 2 College of Environmental Science and Engineering, Ocean University of China, Qingdao, 266100 China; 3 Breast Disease Center, the Affiliated Hospital of Qingdao University, Qingdao University, Qingdao, 266000 China; 4 Department of Pathology, the Affiliated Hospital of Qingdao University, Qingdao, 266000 China; 5 Department of General Surgery, Qingdao Eighth People’s Hospital, Qingdao, 266041 China; 6 Department of Medical Oncology, Qingdao West Coast New Area Central Hospital, Qingdao, 266555 China

**Keywords:** Nomogram, CD28, Prognosis, Lung cancer, Young patients

## Abstract

**Background:**

The prognosis of lung cancer was found to be associated with a series of biomarkers related to the tumor immune microenvironment (TIME), which can modulate the biological behaviors and consequent outcomes of lung cancer. Therefore, establishing a prognostic model based on the TIME for lung cancer patients, especially young patients with lung adenocarcinoma (LUAD), is urgently needed.

**Methods:**

In all, 809 lung cancer patients from the TCGA database and 71 young patients with LUAD in our center were involved in this study. Univariate and multivariate analysis based on clinical characteristics and TIME-related expression patterns (as evaluated by IHC) were performed to estimate prognosis and were verified by prognostic nomograms.

**Results:**

Both LUAD and lung cancer patients with high CD28 expression had shorter disease-free survival (DFS) (*P* = 0.0011; *P* = 0.0001) but longer overall survival (OS) (*P* = 0.0001; *P* = 0.0282). TIME-related molecules combined with clinical information and genomic signatures could predict the prognosis of young patients with LUAD with robust efficiency and could be verified by the established nomogram based on the Cox regression model. In addition, CD28 expression was correlated with an abundance of lymphocytes and could modulate the TIME. Higher CD28 levels were observed in primary tumors than in metastatic tissues.

**Conclusion:**

TIME-related molecules were identified as compelling biomarkers for predicting the prognosis of lung cancer, especially in a cohort of young patients. Furthermore, CD28, which is associated with poor DFS but long OS, might participate in the modulation of the TIME and has a different role in the prognosis of young patients with LUAD.

## Background

Among all malignancies in the past decade in China, lung cancer has ranked first in morbidity with a 5-year overall survival (OS) rate of 19.8%, which is lower than the global average [1]. Non-small-cell lung cancer (NSCLC) accounts for almost 85% of lung cancer patients [2] and shows a trend suggesting that more young patients (aged less than 45 years) are suffering from this disease, especially lung adenocarcinoma (LUAD) [3]. LUAD is a disease with a high degree of malignancy that results in invasion and metastasis; this contributes to the poor prognosis of patients. Recent studies have provided evidence that young patients with LUAD present unique biological and genomic characteristics [4, 5], which revealed the necessity of establishing a model for predicting the prognosis of young patients with LUAD.

The tumor microenvironment (TME) has been suggested to be a substantial regulator of the biological behaviors of malignancies, especially LUAD [6]. Essentially, CD4+ or CD8+ T cells play an important role in cancer immunity and TME modulation. Cytotoxic CD8+ T cells promote tumor clearance by targeting tumor cells for destruction [6]. The CD28 costimulatory pathway, an essential pathway that can signal for the activation of naïve T cells and might participate in cancer immunity [7], is also required for tumor clearance. With recent developments in cancer immunity studies, tumor immune microenvironment (TIME)-related molecules have been included in the evaluation of the prognosis of LUAD. According to a previously published study, the TIME can be divided into 4 subtypes determined by the levels of molecules and cells related to TIME, including programmed death-ligand 1 (PD-L1) and tumor infiltrating lymphocytes (TILs); these consist of Type I (PD-L1-, TILs-), Type II (PD-L1+, TILs+), Type III (PD-L1+, TILs-) and Type IV (PD-L1-, TILs+) [8]. The phenotype of the TIME is an important biomarker for predicting prognosis and participates in the modulation of the biological activity of LUAD [7]. In addition, CD3, CD8, PD-1, PD-L1 and CD28 are well-studied critical components of the immunoscore and immune checkpoints, but the role of these molecules in the prognosis of young patients with LUAD has not been explained in detail [7–9]. Above all, further study is required to clarify the role of TIME-related molecules in the prognosis of young patients with LUAD; thus, we examined the expression level of these 5 molecules.

As is well known, nomograms are widely used tools for the clinical evaluation of prognosis in malignancies such as gastric cancer [10], breast cancer [11] and lung cancer [12]. Previous work on nomograms mostly focused on the basic characteristics and clinical information of patients but paid less attention to the pathological changes, especially the TIME status. Wang et al. [13] confirmed the important role of TME-related molecules in the prognosis of LUAD in a recent study and established a risk assessment model based on the TME. In this study, we included immune factors in a prognostic model for LUAD and constructed a novel nomogram with robust efficacy for young patients with LUAD to establish a novel method to predict the prognosis of young patients with LUAD and further discuss the essential role of the TIME in LUAD.

## Methods

### Patients

Seventy-one young patients (*n* = 71) with resectable LUAD in our center from March 2013 to June 2016 were identified and included in this study. The inclusion criteria were as follows: a) patient age < 45 years; b) performance status: Eastern Cooperative Oncology Group (ECOG) 0–2; c) resectable disease (Stage I-III) and available postoperative pathology; d) available follow-up information until October 2019; and e) consent for targeted sequencing. The exclusion criteria were as follows: a) patient age > 45 years; b) ECOG > 2; c) presence of metastatic disease; or d) loss to follow-up or refusal to participate. Basic clinical and demographic information was collected from all enrolled patients, including age, sex, postsurgical treatment methods, smoking history and family history. The amplification refractory mutation system (ARMS) method based on examination of a panel of 10 driver genes in LUAD (*EGFR*, *ALK*, *ROS1*, *BRAF*, *MET*, *HER-2*, *RET*, *NTRK1*, *PI3K*, and *MEK1*) was performed to detect the status of *epidermal growth factor receptor* (*EGFR*), *anaplastic lymphoma kinase* (*ALK*) and other common genomic alterations. Patients were regularly followed up after surgery, and recurrence was defined as the presence of lung cancer occurring in any site in the patient. All tumors were staged according to the 2019 American Joint Committee on Cancer (AJCC) TNM staging system for lung cancer. Disease progression was diagnosed by two professional physicians experienced in clinical medical oncology. Hematoxylin and eosin (H&E) staining was used to confirm the diagnosis of LUAD. The patient characteristics are shown in Table 1.

**Table 1:** Basic characteristics and survival analysis of young Chinese lung adenocarcinoma patients

Variables	Group	Total (N = 71) Patient(%)	Stable disease(***N*** = 53)	Progression disease (***N*** = 18)	Univariate analysis	Multivariate analysis
**age**	> 45	0 (0)	0	0	NA	NA
	<=45	71 (100)	53	18		
**gender**	male	17 (23.94)	12	5	NA	NA
	female	54 (76.06)	41	13		
**pathology type**	adenocarcinoma	71 (100)	53	18	NA	NA
	other	0 (0)	0	0		
**TNM stage**	I-II	46 (64.79)	41	5	< 0.0001	0.017
	III	25 (35.21)	12	13		
***EGFR*** **or** ***ALK*** **mutation**	EGFR Exon 19 del	22 (30.99)				
	EGFR Exon 21 L858R	16 (22.54)				
	EGFR Exon 21L861Q	2 (2.82)				
	EGFR Exon 18 G719	3 (4.23)				
	ALK-EML4	10 (14.08)				
	mutated	52 (73.24)	38	14	0.607	0.143
	wild type	19 (26.76)	15	4	(EGFR:0.522; ALK:0.802)	
**smoking history**	smoker	7 (9.86)	4	3	0.354	0.247
	non-smoker	64 (90.14)	49	15		
**CD28 status**	low expression	60 (84.51)	52	8	< 0.0001	0.063
	high expression	11 (15.49)	1	10		
**PD-L1 status**	low expression	19 (26.76)	18	1	0.0382	0.116
	high expression	52 (73.24)	35	17		
**PD-1 status**	low expression	38 (53.52)	31	7	0.3712	0.492
	high expression	33 (46.48)	22	11		
**CD3 status**	low expression	26 (36.62)	11	15	< 0.0001	0.001
	high expression	45 (63.38)	42	3		
**CD8 status**	low expression	19 (26.76)	11	8	0.0105	0.765
	high expression	52 (73.24)	42	10		

In addition, tissues samples from 809 lung cancer patients (*n* = 809), including 472 LUAD patients (*n* = 472), derived from The Cancer Genome Atlas (TCGA) database were included in this study. The mRNA expression levels of the genes selected for this study in primary tumor tissues were obtained for the analysis, and the stage information and other patient characteristics were available via the Human Protein Atlas. This study was approved by the Ethics Committee of the Affiliated Hospital of Qingdao University, and the investigations were carried out following the guidelines set by the Declaration of Helsinki. Written informed consent was obtained from all patients included in the study, and all experiments were carried out in accordance with the National Health and Family Planning Commission of the PRC’s guidelines.

### Immunohistochemistry analysis

Five-micrometer-thick sections were sliced from paraffin-embedded tissues for immunohistochemistry (IHC) analysis. Antigen retrieval was performed by boiling the sections in 10 mM citrate buffer (pH 6.0) for 2 min followed by cooling at room temperature for 20 min. Each section was incubated overnight at 4 °C with the following primary antibodies at the indicated dilutions: CD28 (ab113358), 1:400; CD3 (ab16669), 1:150; CD8 (ab4055), 1:200; PD-L1 (ab205921), 1:400; and PD-1 (ab52587), 1:100 (all from Abcam). Next, the sections were incubated with HRP-conjugated secondary antibody at 37 °C for 30 min, rinsed with TBS for 5 min, counterstained with Harris hematoxylin for 1 min, dehydrated and mounted on coverslips. The investigators evaluated the IHC staining based on the mean optical density (MOD) as previously described in published works [14, 15]. All sections were scanned on NanoZoomer slide scanners (NanoZoomer-XR C12000, Hamamatsu) and viewed with NDP.view software (NDP.view2 U12388–01, Hamamatsu). Five randomly selected fields (200X) viewed by an experienced pathological doctor using NDP.view software. The MOD of each section was calculated according to the average ODs of the five views by Image-Pro Plus 6.0 (Media Cybernetics, Inc.). The ROC curve of each variable was measured by SPSS 23.0 (SPSS, Inc.), and Youden indexes were used to determine the best cutoff values. An experienced pathologist classified the CD3+ and/or CD8+ sections as two types of localization [16]: the center of the tumor (CT) and invasive margins (IMs). We used CD3 to represent total lymphocytes and CD8 for cytotoxic T lymphocytes (CTLs).

Fragments per kilobase of transcript per million fragments mapped (FPKM) was used to calculate the RNA expression levels in tissues derived from the TCGA database, and the other statistical methods used were the same as above. The cutoff values of all the variables determined by Youden indexes are shown in Table S1.

### Statistical analysis

Univariate analysis was performed for the selection of variables that could estimate the relationship between the TIME and survival. Then, multivariate analysis using the Cox proportional hazards model was used with variables with *P* < 0.2 from the univariate analysis to construct our prognostic model [10] and reduce the influence of sample size. All of the processes above were conducted on SPSS 23.0 software (SPSS, Inc.). The “rms” package of R software version 3.1.2 (The R Foundation for Statistical Computing, Vienna, Austria) was to construct the nomograms with the variables identified by Cox regression analysis. The nomogram for disease-free survival (DFS) was based on the data from our center, while the nomograms for OS were based on the TCGA database. Discrimination and calibration were used to validate the nomograms. Harrell’s C-indexes ranging from 0.5 (no discrimination) to 1 (perfect discrimination) were used to verify the discrimination [17], which were visualized with calibration plots [12]. Bootstrap analysis with 1000 repeated samples were used for these analysis. Five variables (*P* < 0.2)—TNM stage, *EGFR* or *ALK* mutation status, CD28, PD-L1 and CD3—were selected for construction of the DFS nomogram for young patients with LUAD. Age, TNM stage and CD28 and TNM stage, CD28 and PD-L1 were involved in the construction of the OS nomograms for LUAD patients and lung cancer patients, respectively. To better present the information from the cohort of young patients with LUAD, we used the yellow peak called “density”, which refers to the patient number in the nomogram.

Harrell’s C-index and the area under the ROC curve (AUC) were used to compare the discriminatory ability between the nomograms and individual variables for DFS and OS [10]. All the figures in our study were produced by R software version 3.1.2 (The R Foundation for Statistical Computing, Vienna, Austria), SPSS 23.0 (SPSS, Inc.) or GraphPad Prism 8.0 software. Pearson correlation coefficients were calculated to determine correlations. Student’s t tests were conducted to obtain *P* values, which were two tailed for all tests. *P* < 0.05 was used to define statistical significance except for the selection of nomogram variables from the univariate Cox analysis.

## Results

### Patient characteristics and survival analysis

Seventy-one young patients with LUAD were selected for inclusion in our study. The basic characteristics of the patients are summarized in Table 1. The mean age was 40.4 years (range 27 to 45 years). In the cohort of young patients with LUAD from our center, we observed obvious heterogeneity between our cohort and the population of lung cancer worldwide [4]; 73.24% of our young patients harbored EGFR or ALK mutations. The three most frequent genomic alterations were deletion mutations of EGFR exon 19 (31%), the L858R point mutation in EGFR exon 21 (23%) and echinoderm microtubule associated protein like 4 (EML4)-ALK fusion (14%), which suggested that young patients have a unique genomic signature compared with that of old patients. Basic information relating to the genomic alterations is shown in Figure S1E. In addition, 472 LUAD patients and 809 lung cancer patients from the TCGA database were also included in our study.

**Fig. 1:**
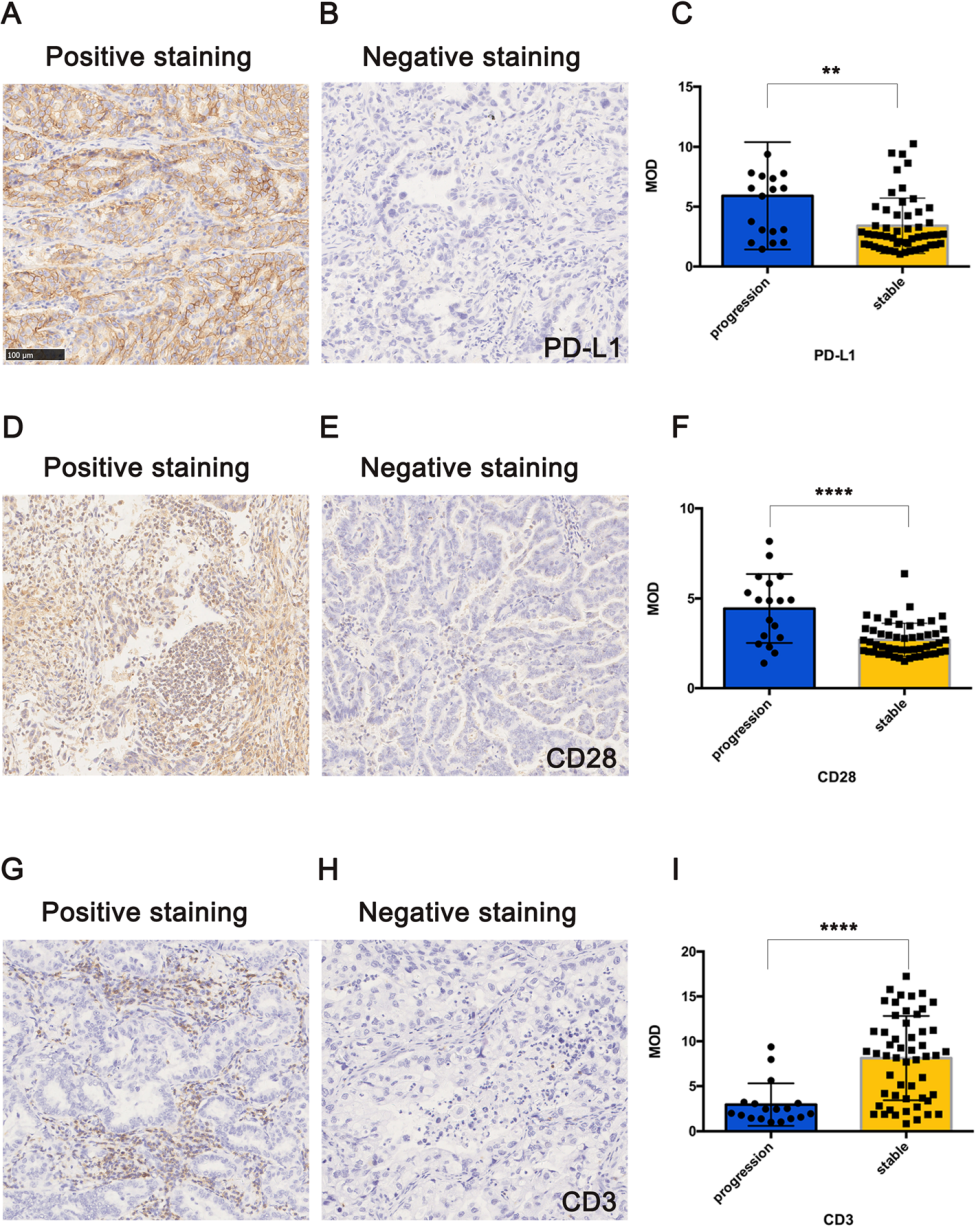
Basic information of young Chinese patients with lung adenocarcinoma (LUAD) and IHC of the tumor immune microenvironment (TIME). **a** Positive PD-L1 staining; **b** Negative PD-L1 staining; **c** The stratification of the MOD of PD-L1 staining between 2 groups of patients; **d** Positive CD28 staining; **e** Negative CD28 staining; **f** The stratification of the MOD of CD28 staining between 2 groups of patients; **g** Positive CD3 staining (center of the tumor); **h** Positive CD3 staining (invasive margins); **i** Negative CD3 staining; **j** The stratification of the MOD of CD3 staining between 2 groups of patients

As shown in Fig. 1, the IHC sections showing staining for the genes stated in the Materials and Methods section were assessed to calculate the MOD. Comparisons of positive and negative staining for PD-L1 and CD28 are shown in Fig. 1a, b, d and e. CD3 staining indicated total lymphocytes, and positive and negative IHC staining of slices is shown in Fig. 1g and h, respectively. In our cohort, there was no significant difference in DFS between the CT and IM groups. The sections showing PD1 and CD8 IHC staining are shown in Figure S1A-D. In this study, we compared the MOD between the progression group (disease recurring before the end of follow-up) and the stable group (no disease recurrence during follow-up) and found a significant difference between them. Figure 1c demonstrates that the progression group had a higher MOD of PD-L1 than did the stable group (*P* = 0.0032); the progression group also had higher CD28 expression (*P* < 0.0001), as shown in Fig. 1f. This finding revealed that PD-L1 and CD28 might be associated with the progressive tendency of LUAD in young patients. However, the MOD of CD3 showed a different distribution, and a higher MOD was detected in the stable group (*P* < 0.0001), as shown in Fig. 1i**,** which indicated that a higher abundance of TILs is probably associated with longer DFS in young patients with LUAD. Although differences in the expression of CD8 and PD1 between the two groups were trending, no statistical significance was confirmed.

**Fig. 2:**
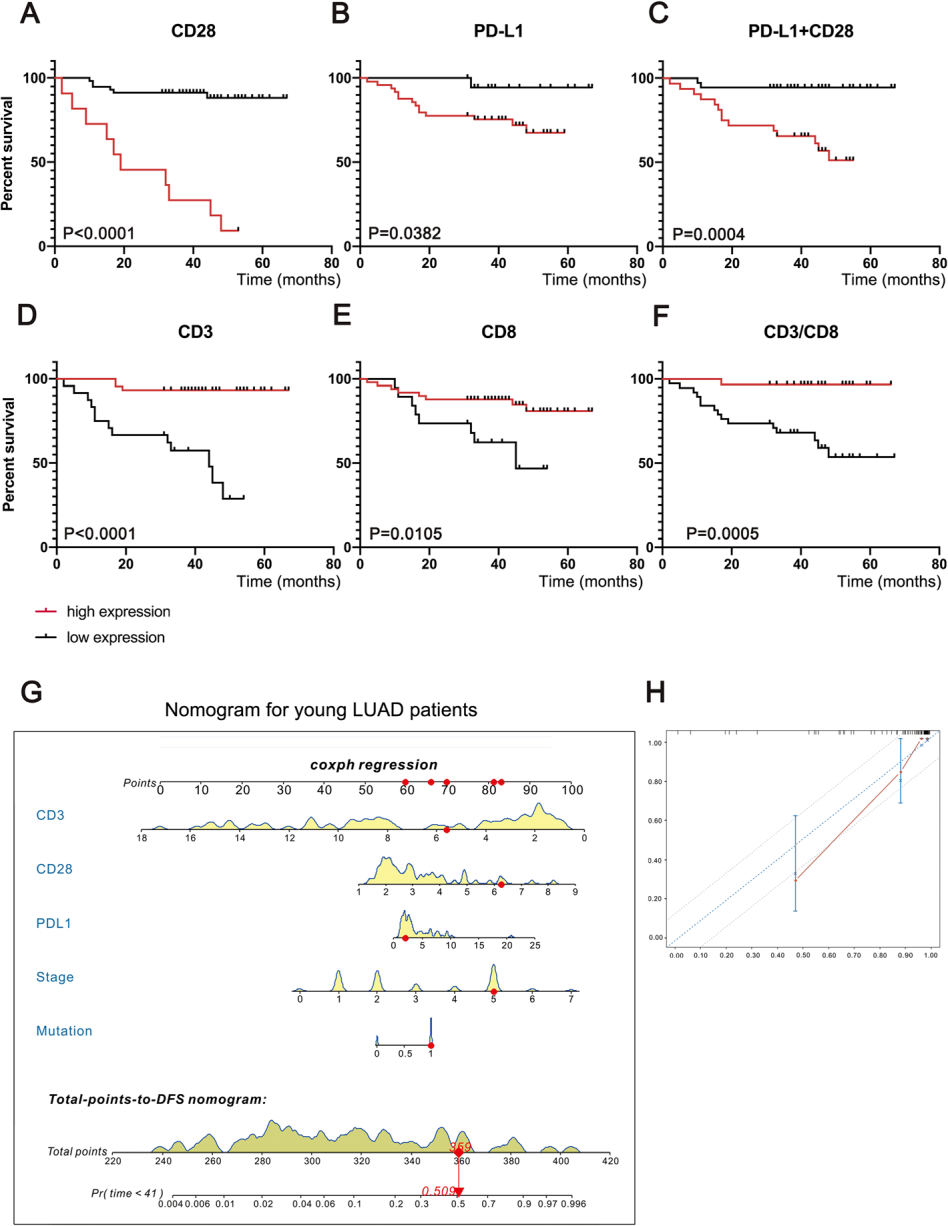
Survival analysis and nomogram for disease-free survival (DFS) of young Chinese patients with LUAD. **a** Kaplan-Meier plot of DFS vs. CD28; **b** Kaplan-Meier plot of DFS vs. PD-L1; **c** Kaplan-Meier plot of DFS vs. CD28 + PD-L1; **d** Kaplan-Meier plot of DFS vs. CD3; **e** Kaplan-Meier plot of DFS vs. CD8; **f** Kaplan-Meier plot of DFS vs. the CD3/CD8 ratio; **g** Nomogram for predicting DFS of young Chinese patients with LUAD; **h** Calibration curve for the DFS nomogram

**Fig. 3:**
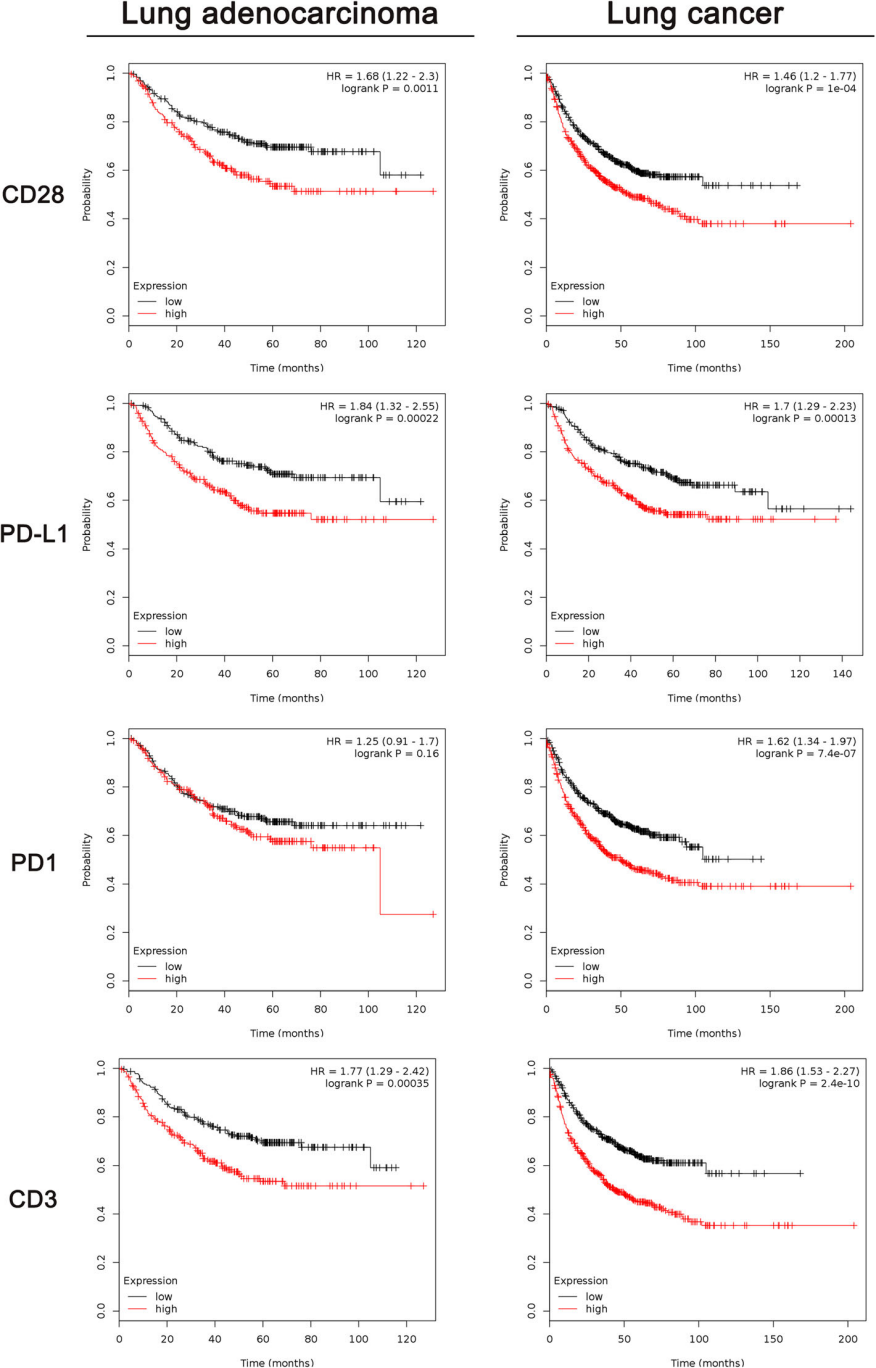
Survival analysis of DFS for LUAD patients and lung cancer patients based on data from The Cancer Genome Atlas (TCGA) database

Survival analysis based on five proteins related to TIME—CD28, PD-L1, PD1, CD3 and CD8—were performed in this study. Thereafter, PD-L1 + CD28 (MOD_PD-L1 + CD28_) and the CD3/CD8 ratio (MOD_CD3/CD8_) were also included in the survival analysis. In young patients with LUAD, CD28 (*P* < 0.0001), PD-L1 (*P* = 0.0382), PD-L1 + CD28 (*P* = 0.0004), CD3 (*P* < 0.0001), CD8 (*P* = 0.0105) and CD3/CD8 (*P* = 0.0005) were all found to be associated with DFS, as shown in Fig. 2a to Fig. 2f**.** Similar to that in the cohort of young patients, the prognosis of all LUAD patients and lung cancer patients could be predicted by CD28 (*P* = 0.0011; *P* < 0.0001), PD-L1 (*P* = 0.0002; *P* = 0.0001) and CD3 (*P* = 0.0004; *P* < 0.0001), as shown in Fig. 3. In addition, CD28 (*P* = 0.016) and CD3 (*P* = 0.0278) correlated with OS of LUAD patients, as shown in Figure S2A-D. For OS of all lung cancer patients, CD28 (*P* = 0.0282) and CD3 (*P* = 0.0032) were found to be compelling biomarkers for predicting prognosis, as shown in Figure S3A-D. Patients with a higher CD28 expression had shorter DFS but longer OS. Elevated PD-L1 expression was associated with poor DFS and OS in lung cancer patients. Moreover, we found that PD-L1 + CD28 might be a better biomarker for DFS in young patients with LUAD. A higher MOD of PD-L1 + CD28 was associated with a shorter DFS in young patients with LUAD. TIL-related variables such as CD3 and CD8 were also associated with DFS among young patients with LUAD. CD3, which represents the total lymphocyte count, was a compelling variable for predicting long DFS in young patients with LUAD but short DFS in all LUAD patients and lung cancer patients. In addition, a higher CD3/CD8 ratio was associated with longer DFS in young patients with LUAD. PD1 and CD3 were not correlated with survival among all LUAD patients or lung cancer patients. The ROC curves of these variables are shown in Figure S1F-J. Assessment of CD28 expression in 809 lung cancer patients suggested that the LUAD group had higher levels of CD28 than did the non-LUAD group (*P* < 0.0001), as shown in Figure S1K.

### The role of the TIME in the prognosis verified by the hazard models

We constructed a nomogram (Fig. 2g) to efficiently predict DFS for young patients with LUAD. The total risk could be measured by the sum of the scores of each variable through the nomogram. The risks of OS for LUAD and lung cancer patients could be evaluated by the nomograms shown in Figure S2E and Figure S3E, respectively. Harrell’s C-indexes revealed that the nomogram for predicting DFS for young patients with LUAD had robust efficiency (C-index = 0.913), but the ability to predict OS for all LUAD patients (C-index = 0.678) or lung cancer patients (C-index = 0.625) was not satisfactory, as indicated in Table 2. Calibration of the nomograms was evaluated by calibration plots, as shown in Fig. 2h, Figure S2F-H and Figure S3F-H; these plots showed that the probabilities of our prognostic models were in agreement with the predicted accuracy on acceptable scales (the dashed lines in the calibration plots correspond to a 10% margin of error), except for 5-year OS in the nomogram of OS in LUAD patients (Figure S2H).

**Table 2:** The discrimination ability of ROC curves and nomograms

	Young LUAD patients		LUAD patients		Lung cancer patients	
	AUC or C-Index	***P*** value^**#**^	AUC or C-Index	***P*** value	AUC or C-Index	***P*** value
**CD28**	0.777	< 0.0001	0.594	0.001	0.556	0.007
**PD-L1**	0.713	0.007	0.531	0.256	0.547	0.025
**PD-1**	0.537	0.639	0.541	0.141	0.545	0.031
**PD-L1 + CD28**	0.81	< 0.0001	0.545	0.107	0.52	0.33
**CD3**	0.832	< 0.0001	0.574	0.008	0.554	0.01
**CD8**	0.506	0.942	0.626	0.345	0.535	0.095
**CD3/CD8**	0.814	< 0.0001	0.531	0.259	0.549	0.018
**Nomograms**	0.913		0.678		0.625	

#: *P* value here refers to statistical significance between AUC (area under ROC curve) area and 0.5

### The role of CD28 in the prognosis and modulation of the TIME in lung cancer

**Fig. 4:**
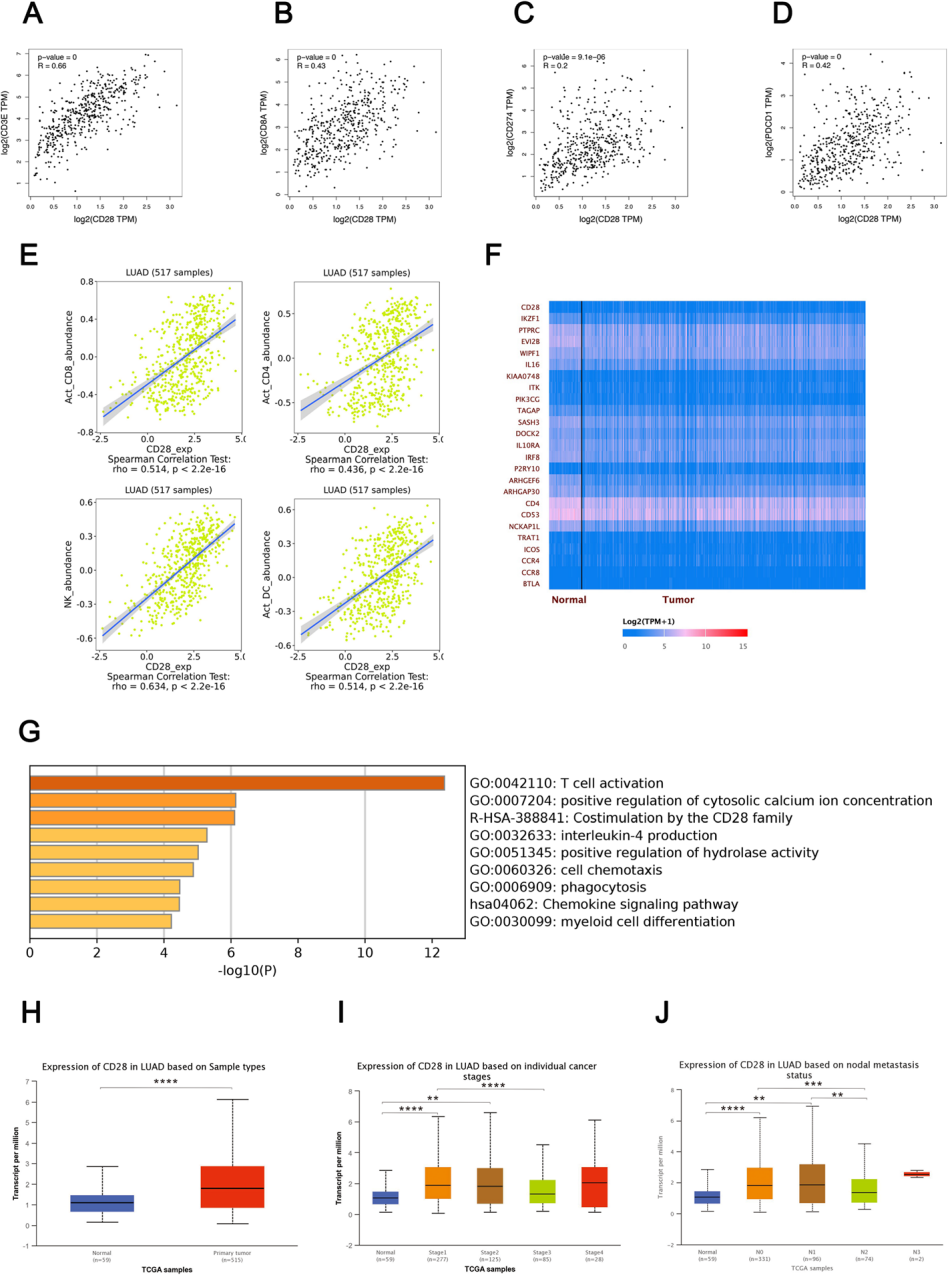
The role of CD28 in the TIME and prognosis of lung cancer. **a** The correlation between CD28 and CD3; **b** The correlation between CD28 and CD8; **c** The correlation between CD28 and PD-L1; **d** The correlation between CD28 and PD1; **e** The correlation between CD28 and total lymphocytes; **f** A heatmap of genes positively correlated with CD28 in LUAD; **g** Enrichment analysis of genes positively correlated with CD28; **h** CD28 expression in LUAD based on sample types; **i** CD28 expression in LUAD based on TNM stage; **j** CD28 expression in LUAD based on lymph node metastasis

In this study, we confirmed the important role of CD28 in the prognosis of lung cancer, especially in young patients with LUAD. As concluded in Table 3, the summary of survival analysis demonstrated that CD28 expression plays different roles in DFS and OS for both LUAD patients and all lung cancer patients. All analysis suggested that CD28 is likely to be associated with poor DFS but good OS. According to samples from the TCGA database, high CD28 expression was observed in primary tumor tissues than in normal control tissues and is associated with an earlier TNM stage and fewer metastatic lymph nodes, as shown in Fig. 4h-j. We further focused on the role of CD28 in the modulation of the TIME in LUAD. Figures 4a-6d describes the correlation between CD28 and other TIME-related molecules through GEPIA based on samples from the TCGA database [18]. Linear correlations based on the Pearson correlation coefficient were confirmed between CD28 and other molecules, including CD3 (R = 0.66), CD8 (R = 0.43), PD-L1 (R = 0.22) and PD1 (R = 0.42). The changes in lymphocytes induced by CD28 were then estimated through TISIDB [19], as shown in Figure S3E. CD28 expression was associated with an abundance of activated CD8+ T cells (*P* < 0.0001), activated CD4+ T cells (*P* < 0.0001), natural killer cells (*P* < 0.0001) and activated dendritic cells (*P* < 0.0001). Based on the data from the samples procured from the TCGA database, we observed that the expression of a group of genes positively correlated (Pearson correlation coefficient > 0.7) with CD28, as listed in Table S2, and a heatmap of these genes is shown in Fig. 4f. Then, enrichment analysis was performed to detect the possible cell signaling pathways related to these genes through “METASCAPE” [20], as shown in Fig. 4g**,** and CD28 is closely associated with the immune-related cell signaling pathways in LUAD, especially T-cell activation. The information for the pathway enrichment is listed in Table S3.

**Table 3:** The different role of CD28 in the prognosis of lung cancer

CD28	LUAD		LUNG CANCER	
	DFS	OS	DFS	OS
**Advantage group**	low expression	high expression	low expression	high expression
**Log-rank** ***P***	0.0011^***^	0.016^*^	0.0001^****^	0.028^*^

## Discussion

Recently, studies have suggested the importance of the TIME in influencing the invasiveness and metastasis of lung cancer [21] and its correlation with tumor heterogeneity [22]. Multiple molecules related to the TIME, such as PD-L1 [23, 24] and PD1 [25], have been used to predict the prognosis of lung cancer patients. In this study, PD1, PD-L1, CD3, CD8 and CD28, which are essential components of the immunoscore and immune checkpoints, were included in the analysis. Given that a younger population exhibits a unique gene expression pattern, the predictive ability of TIME-related molecules in young patients with LUAD needs further study. In young patients, TIME-related molecules were found to be tightly associated with prognosis. However, a single variable does not meet the standard for predicting outcomes related to LUAD, which has been proven to be a disease with tremendous heterogeneity and individual differences among patients. As is well known, nomograms are simple but effective tools for predicting prognosis in medical oncology [26]. Therefore, the prognosis of young patients with LUAD was evaluated through the hazard model based on the nomogram in this study. In our nomogram, multiple TIME-related genes combined with clinical information and genomic signatures were included in the evaluation and displayed satisfactory efficiency in predicting DFS for young patients with LUAD. This result suggested that TIME-related molecules were more likely to be correlated with the prognosis of young patient cohorts than with the whole population of lung cancer patients. In addition, combinations of these variables. Such as PD-L1 + CD28 and the CD3/CD8 ratio, revealed better efficiency in predicting the prognosis of young patients compared with the use of single variables. The coexpression of PD-L1 and CD28, which represent the primary and secondary signals of T cell inhibition via the PD-(L)1 signaling pathway, respectively, correlated with poor prognosis for young patients with LUAD. Furthermore, a higher abundance of CTLs represented by the proportion of CD8+ T cells among total lymphocytes was related to poor outcomes.

A recent study pointed out that the CD28 costimulatory pathway is essentially required for CTL proliferation after PD-1/PD-L1 pathway blockade [27], which indicated that after immune suppression induced by the PD1/PD-L1 pathway, activation of the CD28 costimulatory pathway reverses the suppression status. Therefore, CD28 might serve as a regulator in the TIME of lung cancer. In our work, young LUAD patients with higher levels of CD28 expression or PD-L1 + CD28 had poor DFS, revealing that the high basal levels of CD28 in these patients might exhaust their ability to reverse the tumor immune suppression status which in turn causing the immune escape of tumor cells, resulting in poor DFS consequently. Given that the expression of CD28 is markedly higher in primary tumor tissues than in healthy tissues, T lymphocyte activity might be inhibited in tumor tissue [7], which results in an immune-suppressed microenvironment that participates in endowing cells with oncogenic functions. Therefore, high CD28 expression suppresses the adaptive immune response to cancerous cells and acts as a tumorigenic factor in the early stage of lung cancer development, which is related to the rapid progression of the disease. However, high levels of CD28 expression were related to longer OS, which suggested the different effects of CD28 on the prognosis of lung cancer. With the development of the disease, we observed the loss of CD28 expression in metastatic tissue. This loss might participate in the mechanism of metastasis. The mechanism by which CD28 affects carcinogenesis and cancer development in LUAD needs further study.

Admittedly, our study has some limitations. First, given that young patients with LUAD represent only a small proportion of all lung cancer patients, the sample size of our study was not large enough. However, we followed the instructions of a previously published study [8] and elevated the selective *P* value to 0.2 when selecting variables from the Cox regression models to reduce the influence of sample size on the construction of the models. In addition, several patients harboring EGFR mutations underwent EGFR-TKI treatment after surgery and thus were not included in our model. The influence of postsurgical EGFR-TKI treatment on DFS remains to be further studied.

## Conclusion

In conclusion, the expression of TIME-related molecules, including CD28, PD-L1, CD3 and CD8, is closely associated with the prognosis of young patients with LUAD. CD28, which is associated with poor DFS but long OS, might serve as a novel biomarker for the prognosis of lung cancer, and the different effects of CD28 on lung cancer prognosis should be considered. In addition, CD28 plays an important role in modulating the TIME of LUAD by altering the abundance of immunocytes. Here, we provide a prognostic model based on a nomogram for physicians to establish more individualized follow-up regimens for young patients with LUAD.

## Supplementary information

**Additional file 1: Table S1.** Best cutoffs of all variables. **Table S2.** Genes involved in pathways enrichment analysis. **Table S3.** Information for pathway enrichment. **Figure S1.** Supplementary figures. A) positive staining of PD1; B) negative staining of PD1; C) positive staining of CD8; D) negative staining of CD8; E) the genomic alterations of young LUAD patients; F) ROC of CD28; G) ROC of PD-L1; H) ROC of CD3; I) ROC of PD-L1 + CD28; J) ROC of CD2/CD8;K) the comparison of CD28 expression between non-LUAD and LUAD patients. **Figure S2.** Survival analysis and nomogram for overall survival (OS) based on TCGA for LUAD. A) Kaplan-Meier plot of CD28; B) Kaplan-Meier plot of CD3; C) Kaplan-Meier plot of PD1; D) Kaplan-Meier plot of PD-L1; E) Nomogram for predicting OS in TCGA; F) Calibration curve for the OS nomogram (1-year OS); G) Calibration curve for the OS nomogram (3-year OS); H) Calibration curve for the OS nomogram (5-year OS). **Figure S3.** Survival analysis and nomogram for overall survival (OS) based on TCGA for lung cancer. A) Kaplan-Meier plot of CD28; B) Kaplan-Meier plot of PD-L1; C) Kaplan-Meier plot of PD1; D) Kaplan-Meier plot of CD3; E) Nomogram for predicting OS in TCGA; F) Calibration curve for the OS nomogram (1-year OS); G) Calibration curve for the OS nomogram (3-year OS); H) Calibration curve for the OS nomogram (5-year OS).

## Data Availability

All data generated during this study are included in this published article. The datasets used to generate the data in the current study are available from the TCGA database.

## Abbreviations

NSCLC: Non-small-cell lung cancer
LUAD: Lung adenocarcinoma
TME: Tumor microenvironment
TIME: Tumor immune microenvironment
PD-L1: Programmed death-ligand 1
TILs: Tumor-infiltrating lymphocytes
OS: Overall survival
DFS: Disease-free survival
H&E: Hematoxylin and eosin
TCGA: The Cancer Genome Atlas
ARMS: Amplification refractory mutation system
EGFR: Epidermal growth factor receptor
ALK: Anaplastic lymphoma kinase
EML4: Echinoderm microtubule associated protein like 4
AJCC: AMERICAN Joint Committee on Cancer
IHC: Immunohistochemistry
MOD: Mean optical density
CT: Center of tumor
IM: Invasive margin
CTL: Cytotoxic T lymphocyte
FPKM: Fragments per kilobase of transcript per million fragments mapped
RNA: Ribonucleic acid
AUC: Area under the curve
