# Supplementary material for: The role of CD28 in the prognosis of young lung adenocarcinoma patients
Source: BMC Cancer. 2020 Sep 23;20:910. doi: 10.1186/s12885-020-07412-0 (PMC7510131; doi:10.1186/s12885-020-07412-0)
Supplement: Supplementary file 1 — Additional file 1: Table S1. Best cutoffs of all variables. Table S2. Genes involved in pathways enrichment analysis. Table S3. Information for pathway enrichment. Figure S1. Supplementary figures. A) positive staining of PD1; B) negative staining of PD1; C) positive staining of CD8; D) negative staining of CD8; E) the genomic alterations of young LUAD patients; F) ROC of CD28; G) ROC of PD-L1; H) ROC of CD3; I) ROC of PD-L1 + CD28; J) ROC of CD2/CD8;K) the comparison of CD28 expression between non-LUAD and LUAD patients. Figure S2. Survival analysis and nomogram for overall survival (OS) based on TCGA for LUAD. A) Kaplan-Meier plot of CD28; B) Kaplan-Meier plot of CD3; C) Kaplan-Meier plot of PD1; D) Kaplan-Meier plot of PD-L1; E) Nomogram for predicting OS in TCGA; F) Calibration curve for the OS nomogram (1-year OS); G) Calibration curve for the OS nomogram (3-year OS); H) Calibration curve for the OS nomogram (5-year OS). Figure S3. Survival analysis and nomogram for overall survival (OS) based on TCGA for lung cancer. A) Kaplan-Meier plot of CD28; B) Kaplan-Meier plot of PD-L1; C) Kaplan-Meier plot of PD1; D) Kaplan-Meier plot of CD3; E) Nomogram for predicting OS in TCGA; F) Calibration curve for the OS nomogram (1-year OS); G) Calibration curve for the OS nomogram (3-year OS); H) Calibration curve for the OS nomogram (5-year OS). [file 12885_2020_7412_MOESM1_ESM.docx]

**Table S1. Best cutoffs of all variables.**

| Variables | Cutoffs of MOD | Cutoffs of FPKM (LUAD) | Cutoffs of FPKM (lung cancer) |
| --- | --- | --- | --- |
| CD28 | 4.71 | 0.75 | 0.75 |
| PD-L1 | 1.94 | 0.95 | 3.25 |
| CD3 | 3.28 | 6.55 | 5.85 |
| CD8 | 1.75 | NA | NA |
| PD1 | 1.13 | 0.55 | 1.75 |
| CD28+PD-L1 | 5.83 | NA | NA |
| CD3/CD8 | 2.59 | 1.81 | 1.85 |

**Table S2. Genes involved in pathways enrichment analysis.**

| Genes | Pearson CC |
| --- | --- |
| IKZF1 | 0.83 |
| PTPRC | 0.82 |
| EVI2B | 0.81 |
| WIPF1 | 0.79 |
| IL16 | 0.78 |
| KIAA0748 | 0.77 |
| ITK | 0.77 |
| PIK3CG | 0.77 |
| TAGAP | 0.77 |
| SASH3 | 0.77 |
| DOCK2 | 0.76 |
| IL10RA | 0.76 |
| IRF8 | 0.76 |
| P2RY10 | 0.75 |
| ARHGEF6 | 0.75 |
| ARHGAP30 | 0.75 |
| CD4 | 0.75 |
| CD53 | 0.75 |
| NCKAP1L | 0.75 |
| TRAT1 | 0.74 |
| ICOS | 0.74 |
| CCR4 | 0.74 |
| CCR8 | 0.74 |
| BTLA | 0.74 |

**Table S3. Information for pathway enrichment.**

| **Pathway ID** | **Pathway** | **Log10(P)** | **Log10(q)** |
| --- | --- | --- | --- |
| GO:0042110 | T cell activation | -12.35 | -8.03 |
| R-HSA-388841 | Costimulation by the CD28 family | -6.15 | -3.56 |
| GO:0007204 | positive regulation of cytosolic calcium ion concentration | -6.06 | -3.5 |
| GO:0032633 | interleukin-4 production | -5.18 | -2.74 |
| GO:0051345 | positive regulation of hydrolase activity | -5.02 | -2.61 |
| GO:0060326 | cell chemotaxis | -4.9 | -2.51 |
| GO:0006909 | phagocytosis | -4.48 | -2.15 |
| hsa04062 | Chemokine signaling pathway | -4.47 | -2.15 |
| GO:0030099 | myeloid cell differentiation | -4.21 | -1.95 |


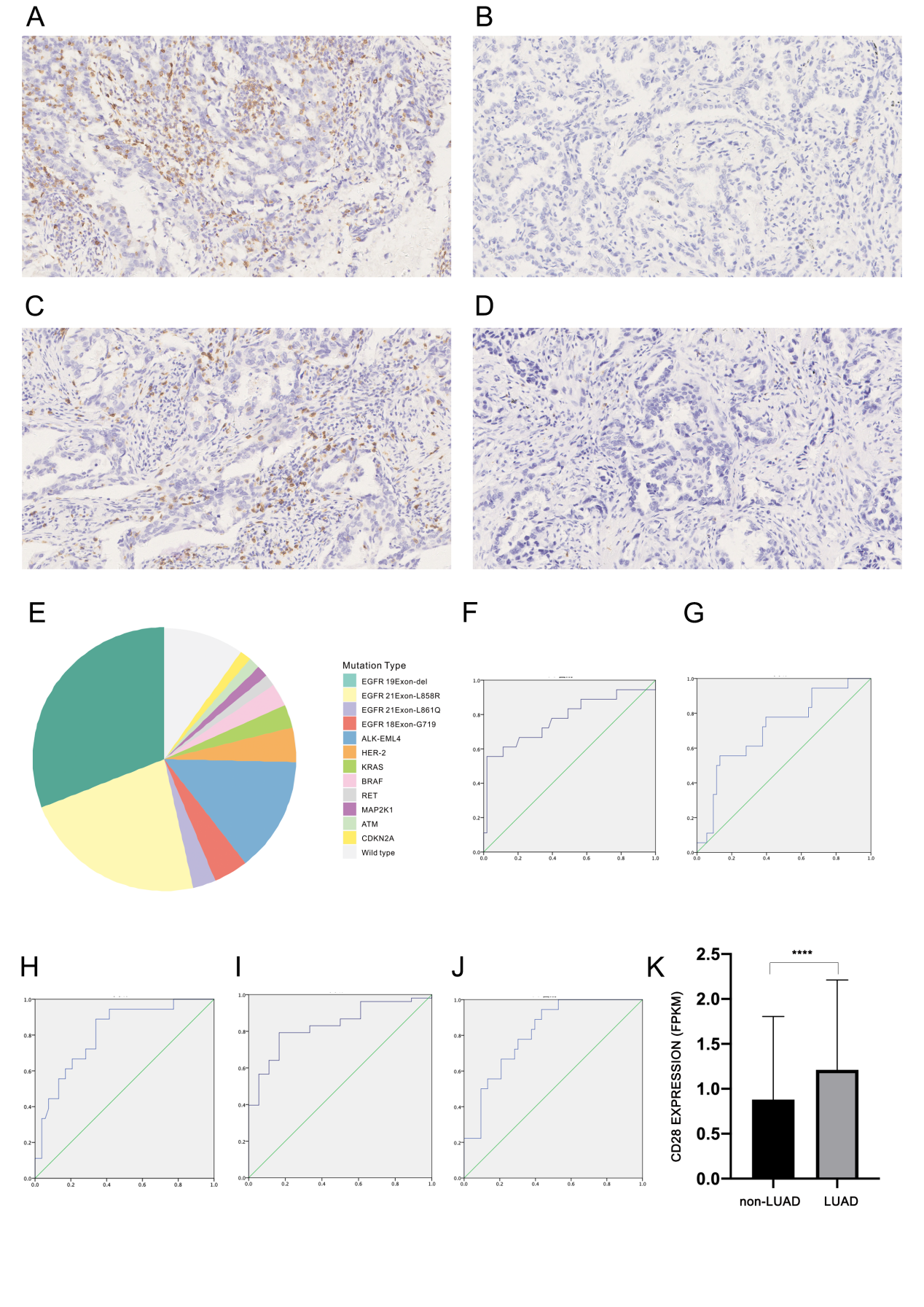


Figure S1. Supplementary figures. A) positive staining of PD1; B) negative staining of PD1; C) positive staining of CD8; D) negative staining of CD8; E) the genomic alterations of young LUAD patients; F) ROC of CD28; G) ROC of PD-L1; H) ROC of CD3; I) ROC of PD-L1+CD28; J) ROC of CD2/CD8;K) the comparison of CD28 expression between non-LUAD and LUAD patients.


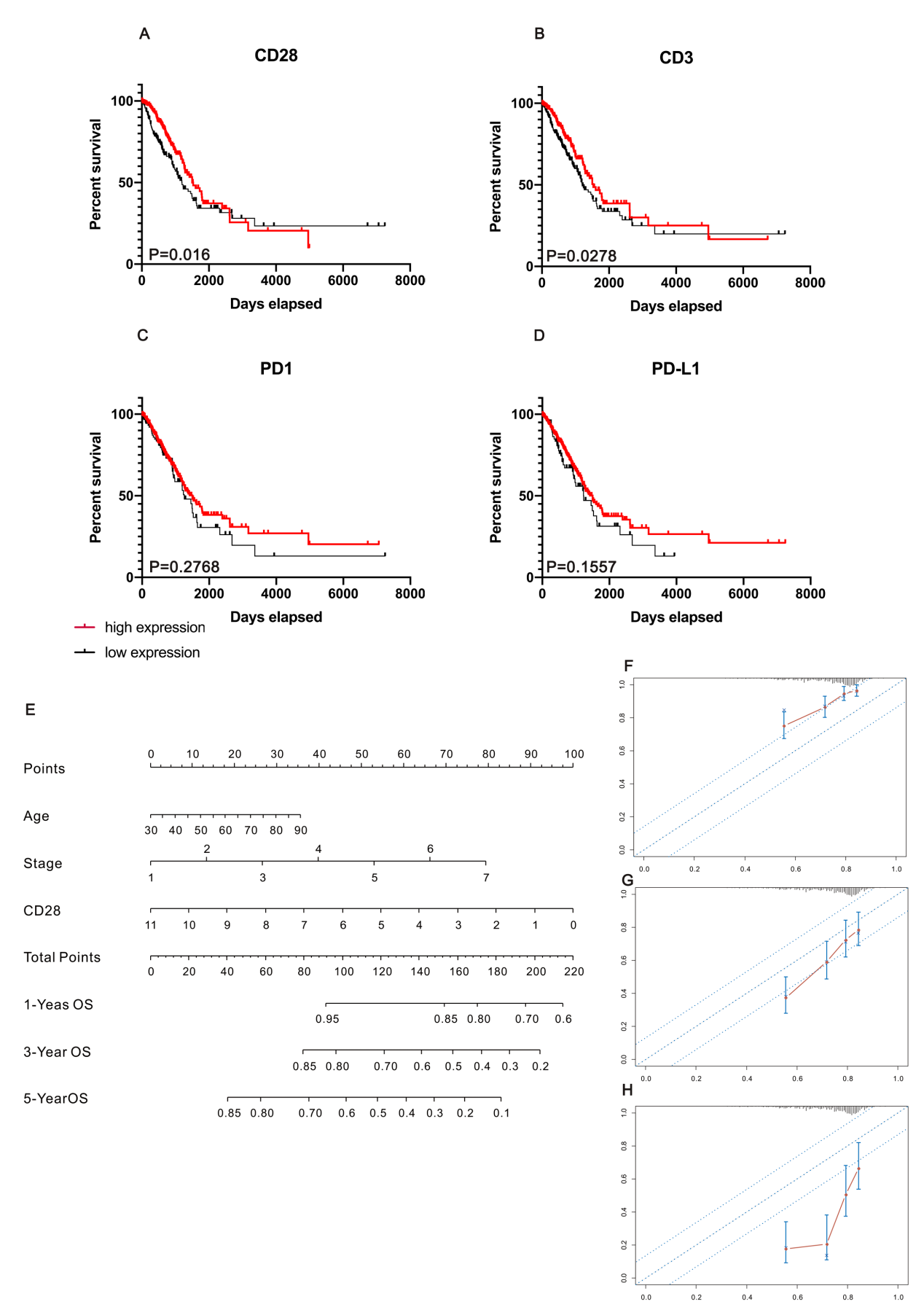


Figure S2. Survival analysis and nomogram for overall survival (OS) based on TCGA for LUAD. A) Kaplan-Meier plot of CD28; B) Kaplan-Meier plot of CD3; C) Kaplan-Meier plot of PD1; D) Kaplan-Meier plot of PD-L1; E) Nomogram for predicting OS in TCGA; F) Calibration curve for the OS nomogram (1-year OS); G) Calibration curve for the OS nomogram (3-year OS); H) Calibration curve for the OS nomogram (5-year OS).


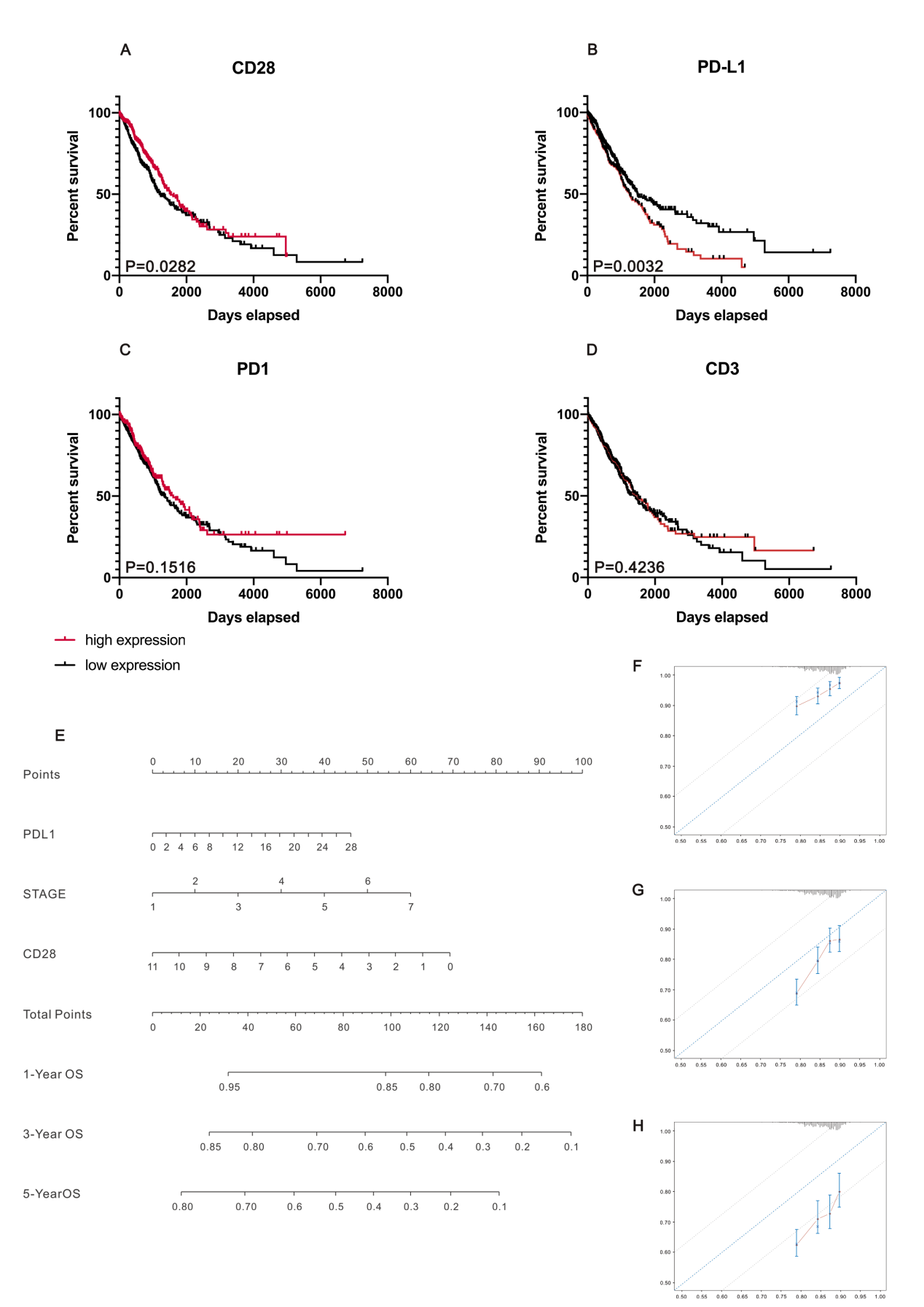


Figure S3. Survival analysis and nomogram for overall survival (OS) based on TCGA for lung cancer. A) Kaplan-Meier plot of CD28; B) Kaplan-Meier plot of PD-L1; C) Kaplan-Meier plot of PD1; D) Kaplan-Meier plot of CD3; E) Nomogram for predicting OS in TCGA; F) Calibration curve for the OS nomogram (1-year OS); G) Calibration curve for the OS nomogram (3-year OS); H) Calibration curve for the OS nomogram (5-year OS).
